# Preventing Early Complications Following Oncologic Breast Surgery: The NDoCaSco Score for Targeted Negative-Pressure Wound Dressing

**DOI:** 10.3390/jpm16060305

**Published:** 2026-06-04

**Authors:** Donato Casella, Juste Kaciulyte, Andrea Bartalini Cinughi de Pazzi, Luca Sanvitale, Alessia Pagnotta, Pietro Maria Ferrando, Alessandro Neri, Marco Marcasciano, Federico Lo Torto

**Affiliations:** 1 Plastic and Reconstructive Surgery Unit, Department of Surgery Sciences, AOU Città della Salute e della Scienza di Torino—Molinette Hospital, 10126 Turin, Italy; donatocasella@gmail.com; 2 Oncologic Breast Surgery Unit, Department of Medicine, Surgery and Neuroscience, University of Siena, 53100 Siena, Italy; bartalinicp@gmail.com (A.B.C.d.P.); neriale65@gmail.com (A.N.); 3 Department of Surgery Sciences, University of Turin, 10126 Turin, Italy; luca.sanvitale97@gmail.com; 4 Plastic and Reconstructive Surgery Unit, Department of Surgical Sciences, Umberto I Hospital, Sapienza University of Rome, 00161 Roma, Italy; pagale@me.com; 5 Plastic Surgery Department, AOU Città della Salute e della Scienza di Torino—San Lazzaro Hospital, 10126 Turin, Italy; pmferrando@hotmail.it; 6 Plastic and Reconstructive Surgery Unit, Department of Experimental and Clinical Medicine, “Magna Graecia” University of Catanzaro, 88100 Catanzaro, Italy; 7 Plastic and Reconstructive Surgery Unit, Department of Medical, Oral and Biotechnological Sciences, “G. D’Annunzio” University of Chieti-Pescara, 66100 Chieti, Italy; federico.lotorto@unich.it

**Keywords:** negative-pressure wound dressing, breast cancer, breast reconstruction, post-operative complication, prevention

## Abstract

**Background:** Thanks to its capacity to increase wound healing, NPWD (Negative-Pressure Wound Dressing) showed promising results in breast surgery. The authors developed the NDoCaSco system for select patients that may benefit the most from NPWD after breast oncologic surgery, aiming to improve outcomes in patients at risk for wound dehiscence and breast reconstruction failure. **Methods:** Patients scheduled for breast oncologic surgery were enrolled between 2022 and 2023. Surgical wound dressing was selected prior to assessing the risk for post-operative complications with the NDoCaSco. Low-risk patients (NDoCaSco score: 15–21) received traditional compressive dressing, while moderate- (NDoCaSco: 8–14) and high-risk (NDoCaSco: 0–7) patients received short-term or long-term NPWD, respectively. **Results:** Healing time and outcomes were compared to a retrospective control group that underwent the same surgeries between 2019 and 2021 and received traditional compressive wound dressing in all cases. The study population included 739 patients with an average age of 62.3 years (range, 29–95) and a mean BMI of 25.2 kg/m^2^ (range, 16–46). Breast-conserving surgery was performed in 437 cases, and 302 received mastectomy with implant-based reconstruction. A total of 152 patients scored medium (140 cases) or low (12 cases) NDoCaSco and received NPWD. Post-operative complications’ incidence, healing time, and drain removal time were lower in the study group, while scar quality was consistently improved with NPWD when comparing the two middle-risk groups. **Conclusions:** NDoCaSco helped in identifying patients who benefit the most from NPWD, achieving faster healing and reduction in outpatient visits and hospital admissions, leading to a lower expenditure of resources.

## 1. Introduction

Breast conservation surgery (BCS) reaches equivalent survival rates to mastectomy and has become the first choice in the majority of cases nowadays [1,2,3,4]. When BCS is not applicable, mastectomy is indicated. In prepectoral implant-based breast reconstruction (IBR) after mastectomy, flaps necrosis represents a dreadful event that leads to implant loss and reconstruction failure in about 10–30% of cases [5,6,7]. Complications following BCS or mastectomy may jeopardize breast reconstruction and surgical outcomes, and they may delay adjuvant oncologic therapies. It is crucial to prevent these circumstances. Main patient- and surgery-related risk factors, such as old age, smoking habits or some comorbidities, are well known [8]. An accurate pre-operative patients’ assessment may help to select the most suitable preventive measures to lower complications’ incidence. One of these is Negative-Pressure Wound Dressing (NPWD) [9,10], which shows potential in reducing post-surgical complications and their economic burden in breast surgery [11,12,13]. Following this trend, the authors of the current retrospective study developed an assessment score to outline the patients scheduled for oncologic breast surgery that may benefit the most from post-operative NPWD. They aimed to standardize the NPWD application in order to maximize the success rate even in patients considered at risk for breast reconstruction failure.

## 2. Materials and Methods

Between 2022 and 2023, patients aged 18 years or older with a breast cancer diagnosis met the basic inclusion criteria. Only cases willing and eligible to undergo BCS or mastectomy followed by IBR were admitted to the study. The presence of BRCA mutation diagnosis was considered an exclusion criteria, due to substantial differences in this population when compared to breast cancer-affected patients, like average age and comorbidities [14]. Prior to mastectomy, patients were evaluated according to the PreBra score to select the safest reconstructive procedure [8]. When feasible, prepectoral DTI (Direct-to-Implant) was performed [15,16] In medium-risk patients, with a PreBra score between 5 and 8, who were still feasible for subcutaneous implant placement despite a few risk factors such as thinner flaps or some comorbidities, a prepectoral tissue expander was placed [17]. In high-risk cases with poor PreBra score (0–4) that presented high risk for breast reconstruction complications due to their anamnesis and/or thin mastectomy flaps, submuscular 2-stage reconstruction was favored [18].

When BCS was indicated, oncoplasty surgeries were planned, assessing pre-operative breast asymmetries, hypertrophy, ptosis and cancer localization. In all cases that presented doubtful perfusion of the nipple–areola complex, mastectomy flaps, or local pedicled flaps in BCS, an intra-operative indocyanine green fluorescence exam was performed. The presence of one or more not-perfused areas influenced the possibility of removing them and the choice of post-operative wound dressing.

In all cases, one vacuum drain was positioned. Wound dressing was selected prior assessing the risk for post-operative complications thanks to the NDoCaSco algorithm (Table 1). The title of the score is an acronym that combines the NPWD (N) with the first author’s initials (DoCa), who conceptualized the score (Sco). Also, it represents word play in Italian slang, where “’n do’ casco” means literally “where do I fall” and it is used to say: “what do I do now that I have a problem”.

The score evaluates 11 domains that have been linked to an increased risk for wound dehiscence, tissue suffering and necrosis. While most of the domains have clear association with wound healing failure, a few of them may deserve some further explanation [19,20,21,22,23,24,25,26,27]. High BMI in particular has been given 1 point due to the fact that it represents a well-known risk factor for surgical complications, with every unit of its increase being associated with a 7.9% rise in the odds of breast reconstructive failure [28]. According to the literature and the authors’ experience, low BMI is associated with an even higher risk of surgical complications, due to poor skin and soft tissue quality, so it has been given 0 points in the current study [29,30,31,32]. Similarly, old age has been linked to a perilous wound healing process due to compromised microvascular circulation and poor soft tissue quality, which brings increased risk of skin flaps damage during mastectomy [33]. This risk increases with age and in particular with the occurrence of menopause in women due to hormonal changes that affect tissue tropism, so the age domain has been evaluated with different scores dividing adults (<50 years) from older adults after menopause (>50 years) and the elderly (>70 years) [34].

At the end of the NDoCaSco evaluation, each patient scores from 0 to 21, with lower scores associated with major healing failure risk.

**Figure 1:**
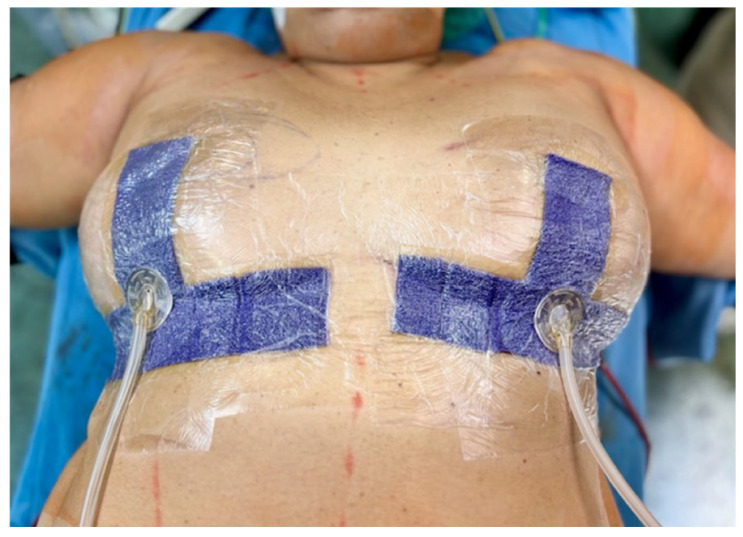
In the current study, the NDoCaSco algorithm was used to select post-operative wound dressing by assessing the risk for post-operative complications. Patients presenting as moderate- (NDoCaSco: 8–14) and high-risk (NDoCaSco: 0–7) received short-term preventive or long-term NPWD, respectively. The image shows an example of bilateral NPWD (−125 mmHg) applied directly in the surgery room, immediately after surgery.

**Figure 2:**
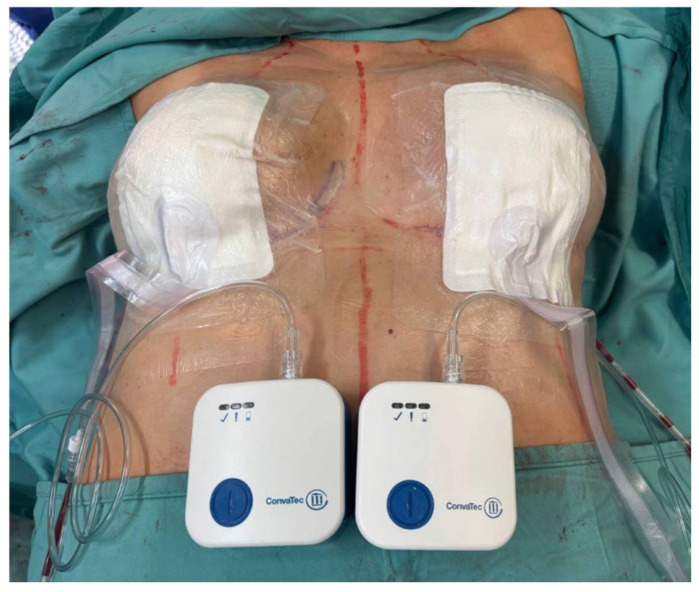
The authors used different types of NPWD, which were chosen based on breast shape and surgery performed and finally depending on which device was at disposal at the moment. The picture shows an additional example of bilateral NPWD that works at −75 mmHg.

Low-risk patients (NDoCaSco score: 15–21) received traditional compressive wound dressing, while moderate- (NDoCaSco: 8–14) and high-risk (NDoCaSco: 0–7) patients received short-term preventive or long-term NPWD, respectively (Table 2, Figure 1 and Figure 2). Moderate-risk cases in particular underwent one cycle of NPWD for 7 days only, with no wound dressing changes in between and followed by traditional plane wound dressing after. Patients considered high-risk instead received NPWD for 14 days, with one wound dressing change in out-patient ambulatory care on day 7. Once the NPWD was removed, traditional plane wound dressing was applied when required.

During the study time-span, various NPWD devices were at disposal in the Unit, and the surgeons chose them according to breast shape and surgery performed and finally depending on their personal preference and on which device was at disposal (see Supplementary Material—Video S1). All the devices shared the same settings: continuous suction with a limited range of negative pressure corresponding to 75–125 mmHg (high Negative-Pressure Wound Dressing). This was to limit any possible bias due to the apparent heterogeneity of the devices used.

The retrospective control group consisted of 752 patients who were treated with BCS or mastectomy and IBR from 1 August 2019, to 31 December 2021, in the same Unit, using the same selection criteria of the study group. This control group underwent retrospective NDoCaSco assessment, but they all received a traditional compressive wound dressing.

Follow-up visits were scheduled after 1 month, 3 months, and 6 months and once per year. During follow-up, healing time, post-operative complications, scar appearance and cancer recurrence rates were recorded. In particular, scar quality was assessed blindly to treatment allocation. For the assessment, the VAS scale was used to evaluate 4 scars’ characteristics (pigmentation, vascularity, pliability, height), and results ranged from 0 to 13, with lower scores related to better scarring.

The NDoCaSco system was based on the data collected in the literature and in the authors’ experience in breast reconstruction published previously. This assessment score takes into consideration relevant patient-related pre-operative and intra-operative risk factors that may potentially influence surgical wound healing in breast surgery. The NDoCaSco predictive model was then tested in the current study, applying it to choose the type of wound dressing in a group of patients. The results were compared to a retrospective evaluation group, and statistical analyses were conducted using SPSS software Version 30 (IBM Corp., Armonk, NY, USA), and all Pearson’s chi-square and the Student’s *t*-tests were considered significant with *p* < 0.05. The study was approved by the Scientific Committee of the Department of Medicine, Surgery, and Neurosciences of the University of Siena. No approval from the Ethics Committee was required, as the devices used were already approved for clinical practice and no modifications were made to standard treatment protocols in the study.

## 3. Results

From 1 January 2022, to 31 December 2023, 739 patients were included in the current study (Table 3).

Table 4 lists the characteristics of the surgical procedures and the subsequent wound dressings applied. Conservative surgery was carried out in 437 cases, and 302 patients received mastectomy. Skin flap perfusion was evaluated with the use of indocyanine green fluorescence technology, and areas with scarce perfusion were resected when possible.

**Figure 3:**
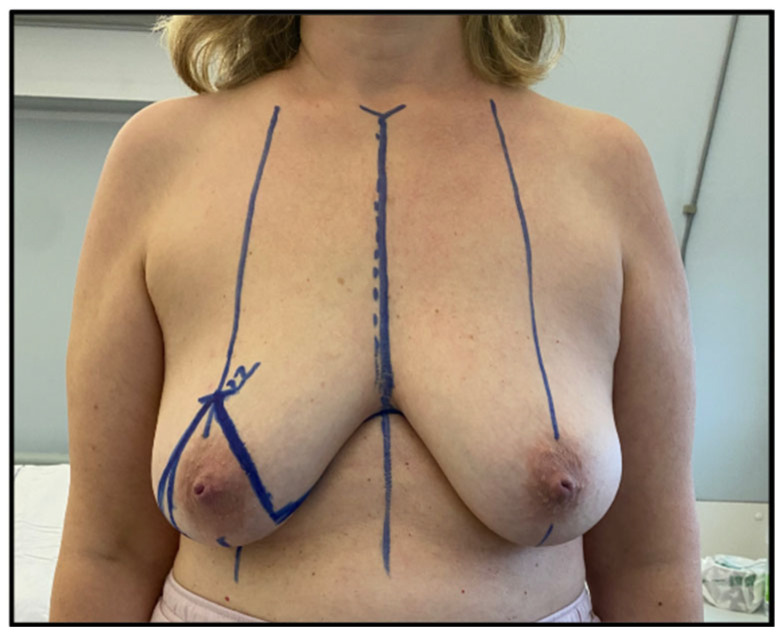
Case 1, pre-operatory picture. This 41-year-old patient presented with cancer affecting her right breast that had undergone neoadjuvant chemotherapy. She had breast ptosis (degree III), so a skin-reducing mastectomy was scheduled. Her NDoCaSco result was 9, and 7-day NPWD was planned after surgery.

**Figure 4:**
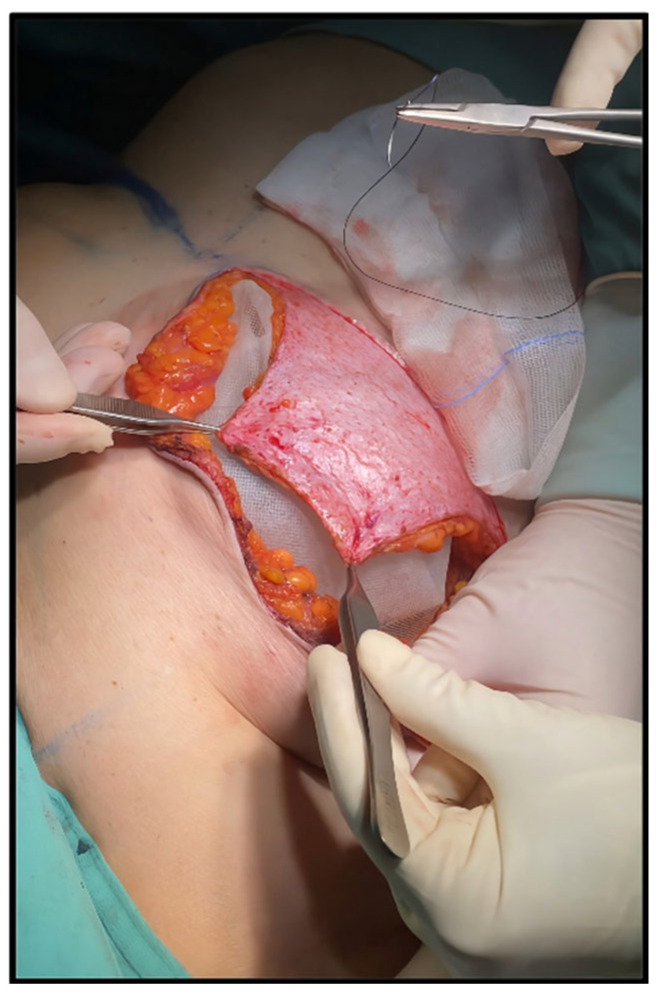
Case 1, intra-operative picture. The patient presented a PreBra score of 8, and prepectoral IBR was carried out by positioning a tissue expander enveloped in synthetic mesh and covered with a dermal flap in the lower quadrants.

**Figure 5:**
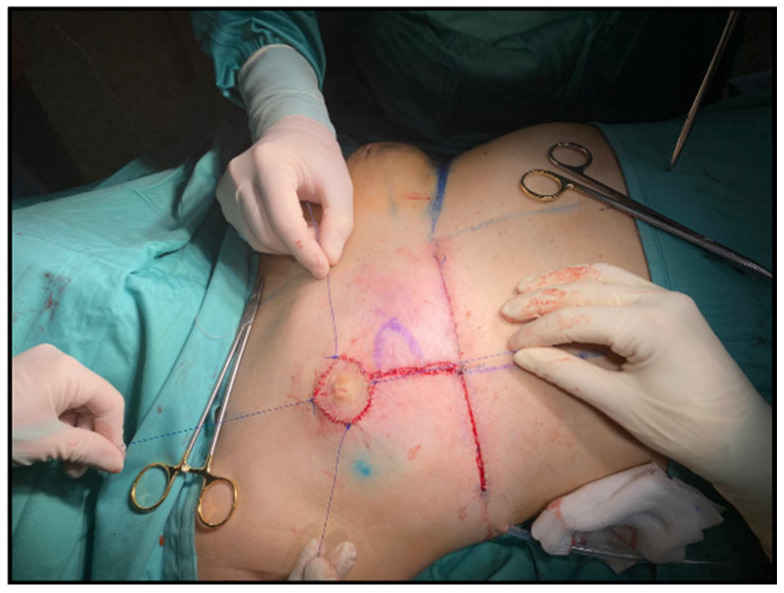
Case 1, intra-operative picture. The intra-operative skin flaps’ perfusion assessment with indocyanine green fluorescence exam showed good perfusion of mastectomy flaps but a scarce perfusion of the nipple–areola complex (NAC) itself. In these cases, the authors chose to detach the NAC and re-implant it as a free graft. The picture shows the re-implanted NAC graft ready to be compressed with a moulage made of gauzes.

**Figure 6:**
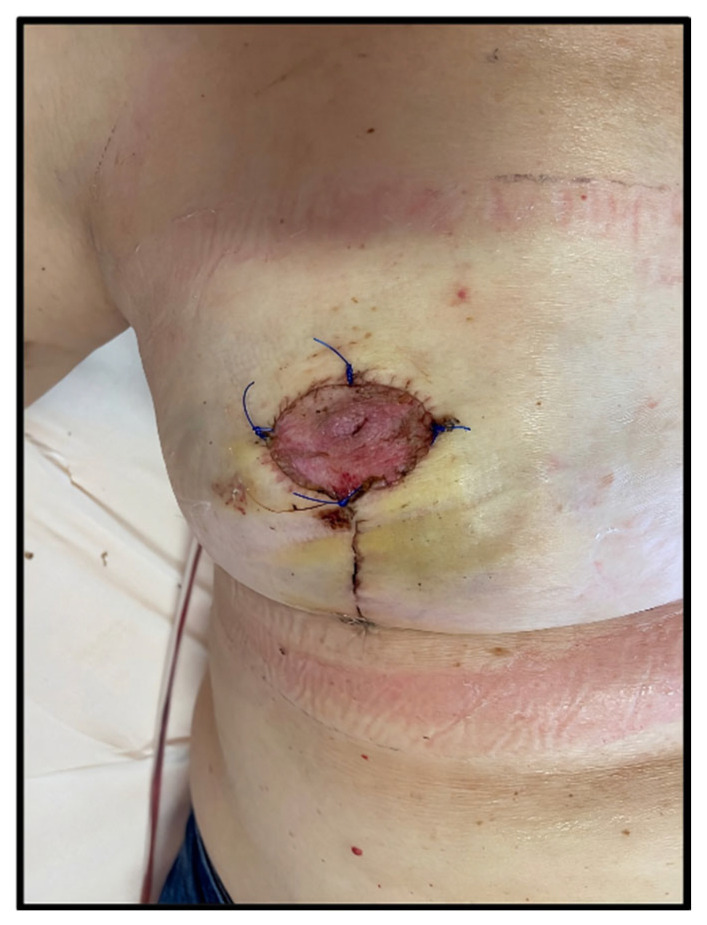
Case 1, early post-operative picture. Seven days after surgery, both NPWD and compressive moulage over the NAC were removed, showing underlying tissues’ vitality. In the picture the drain is visible, ready to be removed as well.

**Figure 7:**
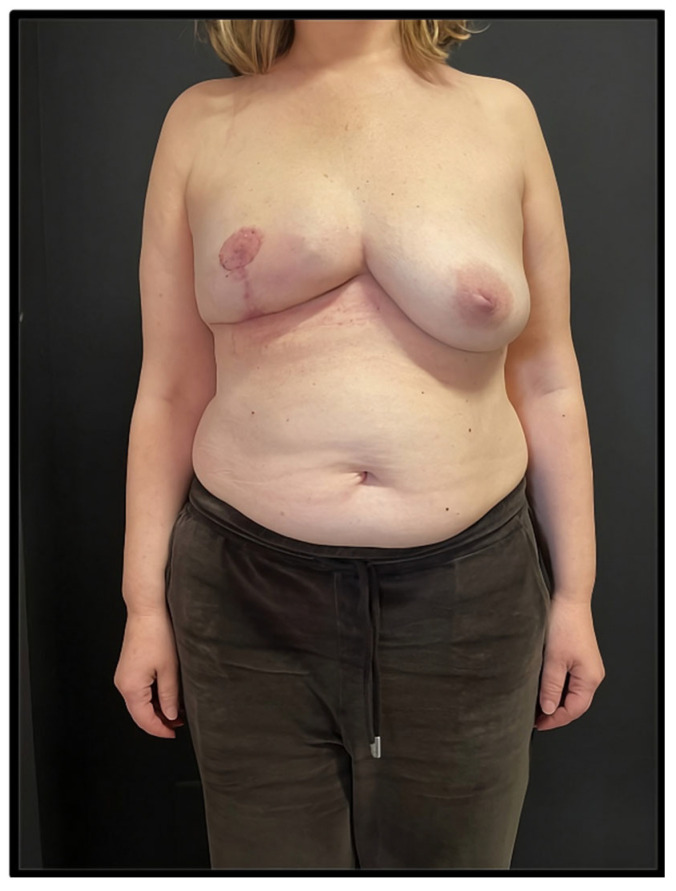
Case 1, post-operative picture. One month after surgery, the patient shows complete wound healing. No complications occurred, and the patient was able to start adjuvant chemotherapy with proper timing.

**Figure 8:**
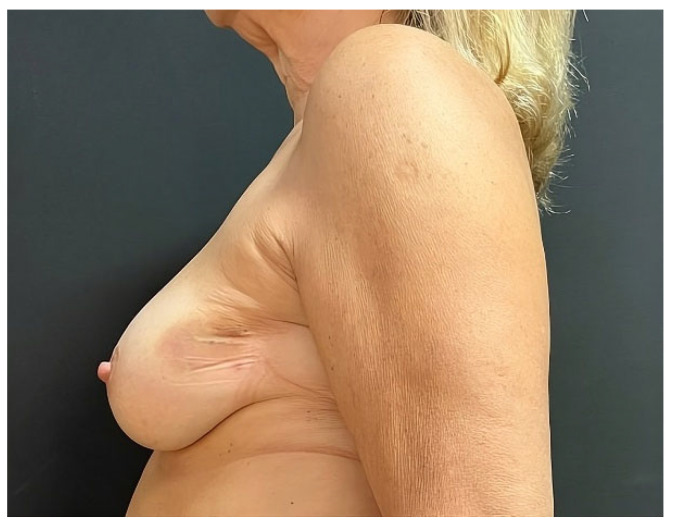
Case 2, pre-operatory profile picture. This 62-year-old woman, a smoker with a history of previous corticosteroid therapies, presented with cancer affecting her left breast’s central-outer quadrant. The tumor was 1.5 cm wide, and BCS with immediate reconstruction with a pedicled LICAP flap was scheduled in order to preserve breast symmetry.

**Figure 9:**
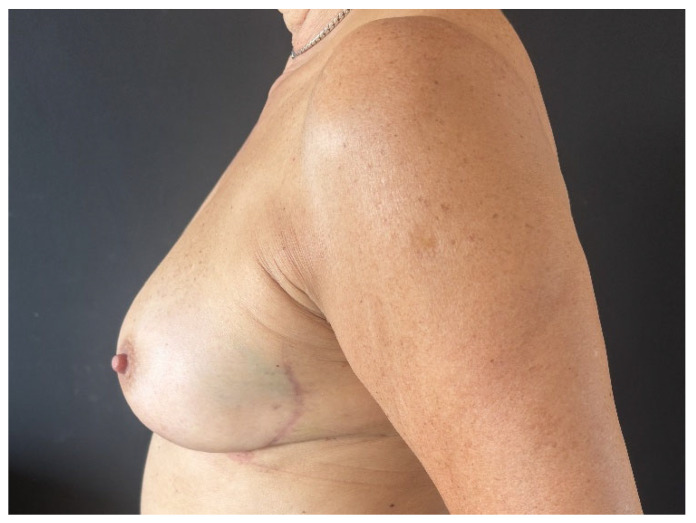
Case 2, post-operatory profile picture taken at the 1-year follow-up, after radiotherapy. The patient’s NDoCaSco value was 14, so NPWD was applied immediately after surgery and kept for 7 days. The patient reached complete healing in 13 days, and no complications occurred. At the 1-year follow-up, the scar was pliable and plane with no retractions. Due to persistent hyperpigmentation, it was evaluated with a score of 5 according to the VAS scale.

All patients were evaluated with the NDoCaSco. High results (15–21) were found in 79.4% of cases (587), and simple compressive wound dressing was positioned. One hundred and forty (18.9%) scored from 8 to 14, and NPWD was applied for 7 days (Figure 3, Figure 4, Figure 5, Figure 6, Figure 7, Figure 8 and Figure 9).

Finally, 12 (1.6%) patients scored a poor result (0–7), and NPWD was positioned for 14 days, with one intermediate wound dressing change in out-patient ambulatory care.

The average post-surgical follow-up period was 22 months (range, 12–50 months), and outcomes were compared to the retrospective control group that accounted for 752 patients, all treated with traditional compressive wound dressing. Table 5 and Table 6 show the control group characteristics and the surgeries performed, evidencing the comparability with the study population.

Postoperative complications were registered in 183 cases (26.2%) in the study group and in 245 cases (32.6%) in the control group. Of those, major complications happened in 50 cases (8.1%) in the study group and in 71 cases (9.4%) in the control group. Overall, patients that underwent NPWD had drains removed quicker (average: 8 days) than the patients with classic wound dressing (average: 10 days). Only healing time, considered as days to complete stitches and wound dressing removal, presented a statistically significant difference between the study (16.5 days) and control group (19.9 days).

Both study and control groups were divided into three subgroups each, according to the NDoCaSco results (high-, medium- and low-risk subgroups), and post-surgical complications and wound healing time were compared within them (Table 7).

Subgroups S1 (587 patients) and C1 (594 patients) consisted of the low-risk population in the study and control groups, respectively. Minor complications were overall lower in S1, although the difference was not statistically significant, likely due to the large sample size relative to the total number of minor complications. Major complications were lower in S1 as well, with no statistical significance. Average healing time was 18.8 days in the retrospective C1 and 16.4 days in S1. This two-day difference in healing time was found to be statistically significant (*p* < 0.05).

Medium- (S2 and C2) and high-risk subgroups (S3 and C3) presented a similar scenario: minor and major complications had lower incidence in the study subgroups, with no statistical significance, while average healing time was significantly shorter in S2 and S3.

No local nor systemic breast cancer recurrence was reported in the current series.

Scar quality was assessed 1 year after surgery, in a blinded way by two authors that did not perform the surgeries. For scar quality evaluation, the Vancouver Scar Scale (VAS) was used [35], giving a score that ranges from 0 to 13, with lower scores related to better scarring. Scar quality was consistently higher in the middle-risk subgroup S2 when compared to C2, which showed an average VAS of 5.3 and 7.1, respectively.

## 4. Discussion

NPWD stimulates microvascular blood flow by increasing angiogenesis. In this way, wound edge perfusion and stable oxygen levels are secured [10,11,12,36], while incidence of seroma, hematoma and infection decreases [37,38,39]. Moreover, NPWD reduces lateral tension forces over the wound and improves scar quality in both appearance and histochemical properties [40,41].

NPWD has shown promising results when applied to breast surgery, with consistent reduction in major complications’ incidence in both DTI and two-stage reconstructions, proving its cost efficacy as an adjunctive benefit [13,42,43,44,45]. Indeed, a complication that leads to wound healing delay up to breast reconstruction failure implies multiple hospital attendances and readmissions with adjunctive surgeries, bearing significant costs [13]. Being more expensive than traditional wound dressing, NPWD shows cost efficacy mostly when applied to cases where high risk of surgical failure justifies its increased price [46,47].

In the current retrospective study, the authors aimed to narrow the application of preventive NPWD to a specific group of patients that were selected according to their risk factors for post-operative complications. A previous study described a simple patients’ selection for NPWD, based on risk factors for post-operative seroma and wound dehiscence [48]. Following this lead, the authors developed the NDoCaSco system to identify the cases that would benefit most from NPWD. Relevant patient characteristics and previous therapies were inserted in a simple scoring system that assures an objective assessment, with easy reproducibility, inter-observer reliability and external validation. The same risk assessment score was applied retrospectively to a control group that underwent the same type of surgeries in previous years and received standard compressive wound dressings.

Overall, post-operative complications’ incidence and time for drain removal were lower in the study group. When comparison was carried out between the subgroups, the study subgroups showed better outcomes in terms of complications and scar quality. Healing time was significantly shorter in the study subgroups, and scar quality was consistently improved with NPWD when comparing the two middle-risk subgroups.

### Limitations

The limitations of this study include its single-center nature and the retrospective selection of the control group, which may have introduced a Will Rogers phenomenon. Indeed, despite the two groups showing similar characteristics with no statistically significant differences, eventual changes in surgical techniques, perioperative care and patient selection over time may have occurred, giving significant bias risk to the current study. With the NDoCaSco selection system solely being based on literature research and the authors’ experience, the current study aimed to provide a preliminary retrospective analysis of its efficacy as a starting point, laying down the basis for the next prospective and multicenter study. This second study will have the aim to give an external, objective validation to the score and the results presented by comparing the outcomes between two prospective groups, thus reducing the consistent bias of the current paper due to the retrospective comparison group.

## 5. Conclusions

When applied preventively based on objective risk prediction models such as the NDoCaSco, NPWD represents a strategic tool for resource optimization. It allows for both significant rationalization of direct and indirect healthcare costs related to postoperative complication management and an improved surgical outcome with faster initiation of any adjuvant therapies. Even more, patients’ satisfaction may increase thanks to lower incidence of complications and less visible scars.

## Figures and Tables

**Table 1:** The NDoCaSco system evaluates individual pre- and intra-operative risk factors for wound healing impairment after breast surgery. Each of the 11 factors receives a score from 0 to 1 or 2. Total patient score varies from 0 to 21.

Risk Factor	Score: 0	Score: 1	Score: 2
Patient’s age	>70	50–70	<50
Diabetes	YES	Hbg < 9 g/dL	NO
Connective tissue diseases	Active	Not active	NO
Corticosteroids therapy	YES	Previous	NO
Smoker	Current smoker	Ex-smoker	Never smoker
BMI	Low: <22	High: >25	Medium: 22–25
Breast ptosis	Grade III	Grade I–II	NO
Previous breast surgery	Major previous breast surgery	Minor previous breast surgery	NO
Previous radiotherapy	Previous breast irradiation	Previous mediastinum irradiation	NO
Chemotherapy	Previous neo-adjuvant CHT	NO	-
Skin flaps’ vitality and thickness	1 or more not perfused, not resectable areas	1 not perfused, resectable area	Complete perfusion

Hbg, Hemoglobin blood level; BMI, Body Mass Index.

**Table 2:** Individual patient scores for NPWD application following breast oncologic surgery and reconstruction.

Score	Post-Operative Wound Management Indications
0–7	NPWD for 14 days.
8–14	Preventive NPWD for 7 days.
15–21	No indication of post-operative NPWD, indication for traditional compressive dressing.

NPWD, Negative-Pressure Wound Dressing.

**Table 3:** Demographic characteristics of the 739 total patients included in the study.

739 Cases	Value (Range or %)
Age	62.3 years (29–95 years)
BMI	25.2 kg/m^2^ (16–46 kg/m^2^)
Diabetes	57 (7.7%)
Connective tissue disorder	2 (0.3%)
Corticosteroid use Active use Past use	44 (5.4%) 4 (0.5%)
Smoking Active smoker Past smoker Never smoker	113 (15.3%) 86 (11.6%) 540 (73.1%)
Breast ptosis I–II° III°	240 (32.5%) 149 (20.2%)
Previous neo-adjuvant chemotherapy	76 (10.3%)
Previous radiotherapy Previous RT over the breast Previous RT over the mediastinum	22 (3.0%) 22 (3.0%) 0
Previous breast surgery Minor breast surgery - Local excision - Minor mastopexy Major breast surgery - QUART - Mastectomy - Reduction mammoplasty - Breast augmentation	46 (6.1%) 24 (3.2%) 23 (3.1%) 1 (0.1%) 22 (2.9%) 21 (2.8%) 1 (0.1%) 0 0

Abbreviations: BMI, Body Mass Index; RT, Radiotherapy; QUART, Quadrantectomy with Axillary lymph node dissection plus Radiation Therapy.

**Table 4:** Baseline characteristics of the 739 surgical procedures performed during the study, the wound dressings applied and the post-operative complications registered during the follow-up.

Characteristic	Total Surgeries: 739
Mastectomy Monolateral Bilateral Breast reconstruction after mastectomy Prepectoral DTI Prepectoral two-stage with TE Submuscular two-stage with TE	302 (40.9%) 299 3 87 (28.8% of mastectomies) 197 (65.2%) 18 (5.9%)
Conservative Surgery Monolateral Bilateral Oncoplasty in monolateral cases Controlateral symmetrization surgery Volume restoration with ICAP flaps	437 (59.1%) 430 7 40 (9.2% of conservative surgeries) 127 (29.1%)
Intra-operative skin flaps assessment Complete perfusion 1 not perfused, resectable area 1 or more not perfused, not resectable areas	550 (74.4%) 60 (8.1%) 129 (17.5%)
NDoCaSco value—wound dressing applied Score 0–7: 14 days NPWD Score 8–14: 7 days NPWD Score 15–21: simple compressive wound dressing	12 (1.6%) 140 (18.9%) 587 (79.4%)
Early minor complications Wound dehiscence Hematoma/Seroma Infection	133 (18.1%) 81 47 5
Major complications Skin necrosis/wound dehiscence Seroma Hematoma Infection	50 (8.1%) 16 10 13 11

Abbreviations: DTI, Direct-to-Implant; TE, Tissue Expander; ICAP, Inter-Costal Artery Perforator; NPWD, Negative-Pressure Wound Dressing.

**Table 5:** Demographic characteristics of the 752 patients composing the retrospective control group.

752 Cases	Value (Range or %)	*p*-Value
Age	62.2 years (28–93 years)	0.8
BMI	25.0 kg/m^2^ (16–47 kg/m^2^)	0.3
Diabetes	35 (4.7%)	0.06
Connective tissue disorder	1 (0.1%)	0.5
Corticosteroid use Active use Past use	46 (6.1%) 6 (0.8%)	0.8
Smoking Active smoker Past smoker Never smoker	142 (18.9%) 99 (13.2%) 511 (68%)	0.08
Breast ptosis I–II° III°	226 (30.1%) 174 (23.1%)	0.3
Previous neo-adjuvant chemotherapy	67 (8.6%)	0.2
Previous radiotherapy Previous RT over the breast Previous RT over the mediastinum	28 (3.7%) 28 (3.7%) 0	0.4
Previous breast surgery Minor breast surgery - Local excision - Minor mastopexy Major breast surgery - QUART - Mastectomy - Reduction mammoplasty - Breast augmentation	51 (6.8%) 21 (2.8%) 21 (2.8%) 0 30 (4%) 28 (3.7%) 2 (0.3%) 0 0	0.7

The last column shows the Pearson correlation coefficient with the same values of the study group. The correlations were considered non-significant, as all the *p*-values were found to be > 0.05. Abbreviations: BMI, Body Mass Index; RT, Radiotherapy; QUART, Quadrantectomy with Axillary lymph node dissection plus Radiation Therapy.

**Table 6:** Baseline characteristics of the 752 surgical procedures and the post-operative complications registered in the retrospective control group.

Characteristic	Total Surgeries: 752	*p*-Value
Mastectomy Monolateral Bilateral Breast reconstruction after mastectomy Prepectoral DTI Prepectoral two-stage with TE Submuscular two-stage with TE	290 (38.6%) 286 4 75 (25.9% of mastectomies) 186 (64.1%) 29 (10.0%)	0.3
Conservative Surgery Monolateral Bilateral Oncoplasty in monolateral cases Controlateral symmetrization surgery Volume restoration with ICAP flaps	462 (61.4%) 455 7 47 (10.2% of conservative surgeries) 115 (24.9%)	0.3
Intra-operative skin flaps assessment Complete perfusion 1 not perfused, resectable area 1 or more not perfused, not resectable areas	545 (72.5%) 86 (11.4%) 121 (16.1%)	0.09
NDoCaSco value—wound dressing applied Score 0–7: simple compressive wound dressing Score 8–14: simple compressive wound dressing Score 15–21: simple compressive wound dressing	11 (1.5%) 147 (19.6%) 594 (78.9%)	-
Early minor complications Wound dehiscence Hematoma/Seroma Infection	174 (23.2%) 114 45 15	-
Major complications Skin necrosis/wound dehiscence Seroma Hematoma Infection	71 (9.4%) 27 13 15 16	-

The last column shows the Pearson correlation coefficient with the same values registered in the study group. In particular, the correlations were assessed for the numbers of mastectomies or conservative surgeries performed and for the intra-operative flaps’ assessment. The correlations were considered non-significant, as all the *p*-values were found to be >0.05. Abbreviations: DTI, Direct-to-Implant; TE, Tissue Expander; ICAP, Inter-Costal Artery Perforator.

**Table 7:** Outcome comparison between the three study sub-groups that received NDoCaSco wound dressing indications (total: 739 cases) and the three retrospective control sub-groups that did not receive NPWD at all (total: 752 cases).

NDoCaSco	Study Population (S): 739	Control Population (C): 752	Minor Early Complications	Major Early Complications	Time to Heal	Scar Quality (VAS Score)
Score 0–7	S3: 12 (1.6%) NPWD for 14 days	C3: 11 (1.5%) Compressive wound dressing	S3			
			Hematoma/seroma: 0 Dehiscence: 2 (16.7%) Infection: 0	Hematoma/seroma: 1 (8.3%) Infection: 1 (8.3%) Flap necrosis: 1 (8.3%)	24.5 (19–35)	9.3
			C3			
			Hematoma/seroma: 1 (9.1%) Dehiscence: 2 (18.2%) Infection: 0	Hematoma/seroma: 2 (18.2%) Infection: 2 (18.2%) Flap necrosis: 2 (18.2%)	35.3 (21–41)	9.8
Score 8–14	S2: 140 (18.9%) NPWD for 7 days	C2: 147 (19.6%) Compressive wound dressing	S2			
			Hematoma/seroma: 13 (9.3%) Dehiscence: 17 (12.1%) Infection: 1 (0.7%)	Hematoma/seroma: 6 (4.2%) Infection: 5 (3.6%) Flap necrosis: 2 (1.4%)	16.5 (12–25)	5.3
			C2			
			Hematoma/seroma: 11 (7.5%) Dehiscence: 39 (26.5%) Infection: 5 (3.4%)	Hematoma/seroma: 10 (6.8%) Infection: 4 (2.7%) Flap necrosis: 6 (4.1%)	23.5 (15–27)	7.1
Score 15–21	S1: 587 (79.4%) Compressive wound dressing	C1: 594 (78.9%) Compressive wound dressing	S1			
			Hematoma/seroma: 34 (5.8%) Dehiscence: 62 (10.6%) Infection: 4 (0.7%)	Hematoma/seroma: 16 (2.7%) Infection: 5 (0.9%) Flap necrosis: 13 (2.2%)	16.4 (13–22)	3.2
			C1			
			Hematoma/seroma: 33 (5.6%) Dehiscence: 73 (12.3%) Infection: 10 (1.7%)	Hematoma/seroma: 16 (2.7%) Infection: 10 (1.7%) Flap necrosis: 19 (3.2%)	18.8 (12–23)	3.4

Abbreviations: NPWD, Negative-Pressure Wound Dressing.

## Data Availability

The original contributions presented in this study are included in the article/Supplementary Material. Further inquiries can be directed to the corresponding author.

## Supplementary Materials

The following supporting information can be downloaded at: https://www.mdpi.com/article/10.3390/jpm16060305/s1. Video S1: The video illustrates the passages to position one of the NPWD devices used in the current study. The procedure is performed immediately after surgery, with the patient still under general anesthesia to assure stillness and wound sterility. It begins with the positioning of the customizable foam over the wounds and its fixing with adhesive transparent film. After practicing a small incision of the dressing, it is connected to the device that produces suction. This device in particular allows it to reach local negative pressure values of −120 mmHg.

## References

[1] Cancer Research, UK Breast Cancer Incidence (Invasive) Statistics. https://www.cancerresearchuk.org/health-professional/cancer-statistics/statistics-by-cancer-type/breast-cancer/incidence-invasive#heading-Zero.

[2] Cancer Register in Italy in 2020. https://www.aiom.it/wp-content/uploads/2020/10/2020_Numeri_Cancro-operatori_web.pdf.

[3] Davies C., Johnson L., Conefrey C., Mills N., Fairbrother P., Holcombe C., Whisker L., Hollingworth W., Skillman J., White P. (2024). Clinical and patient-reported outcomes in women offered oncoplastic breast-conserving surgery as an alternative to mastectomy: ANTHEM multicentre prospective cohort study. Br. J. Surg..

[4] Veronesi U., Cascinelli N., Mariani L., Greco M., Saccozzi R., Luini A., Aguilar M., Marubini E. (2002). Twenty-year follow-up of a randomized study comparing breast conserving surgery with radical mastectomy for early breast cancer. N. Engl. J. Med..

[5] Leff D.R., Bottle A., Mayer E., Patten D.K., Rao C., Aylin P., Hadjiminas D.J., Athanasiou T., Darzi A., Gui G. (2015). Trends in immediate postmastectomy breast reconstruction in the united kingdom. Plast. Reconstr. Surg. Glob. Open..

[6] Martin L., O’donoghue J.M., Horgan K., Thrush S., Johnson R., Gandhi A. (2013). Acellular Dermal Matrix (ADM) assisted breast reconstruction procedures: Joint guidelines from the Association of Breast Surgery and the British Association of Plastic, Reconstructive and Aesthetic Surgeons. Eur. J. Surg. Oncol. (EJSO).

[7] Hultman C.S., Daiza S. (2003). Skin-sparing mastectomy flap complications after breast reconstruction: Review of incidence, management, and outcome. Ann. Plast. Surg..

[8] Casella D., Kaciulyte J., Lo Torto F., Federico M.D., Mori F.L.R., Barellini L.M.D., Fausto A.M.D., Fanelli B.M.D., Greco M.M.D., Ribuffo D.M.D. (2021). “To Pre or Not to Pre”: Introduction of a Prepectoral Breast Reconstruction Assessment Score to Help Surgeons Solving the Decision-Making Dilemma. Retrospective Results of a Multicenter Experience. Plast. Reconstr. Surg..

[9] Buchanan P.J., Kung T.A., Cederna P.S. (2014). Evidence-based medicine: Wound closure. Plast. Reconstr. Surg..

[10] Scalise A., Tartaglione C., Bolletta E., Roberto C., Giovanni N., Marina P., Luca G., Giovanni D.B. (2015). The enhanced healing of a high-risk, clean, sutured surgical incision by prophylactic negative pressure wound therapy as delivered by Prevena Customizable: Cosmetic and therapeutic results. Int. Wound. J..

[11] Kim D.Y., Park S.J., Bang S.I., Mun G.H., Pyon J.K. (2016). Does the Use of Incisional Negative-Pressure Wound Therapy Prevent Mastectomy Flap Necrosis in Immediate Expander-Based Breast Reconstruction?. Plast. Reconstr. Surg..

[12] Murphy J.A., Myers D., Trueman P., Searle R. (2021). Cost-effectiveness of single-use negative-pressure therapy compared with standard care for prevention of reconstruction failure in prepectoral breast reconstruction. BJS Open.

[13] Molska M., Wojciech M., Pieszko K., Cieśla S., Murawa D. (2025). Randomized controlled trial comparing single-use negative-pressure wound therapy (sNPWT) with standard dress-ings during tissue expander-to-implant exchanges. Assessment of risk factors for impaired wound healing and clinical indications for sNPWT. Eur. J. Surg. Oncol..

[14] Wiesmeier A., Prantl L., Zemann F., Silvan E., Vanessa B., Dmytro O., Philipp U., Sophia D., Marc R., Alexandra M.A. (2026). Predictors of Complications in Prophylactic Mastectomy and Direct-to-Implant Breast Reconstruction: A Retrospective, Single-Center Study. J. Clin. Med..

[15] Bernini M., Calabrese C., Cecconi L., Santi C., Gjondedaj U., Roselli J., Nori J., Fausto A., Orzalesi L., Casella D. (2015). Subcutaneous direct-to-implant breast reconstruction: Surgical, functional, and aesthetic results after long-term follow-up. Plast. Reconstr. Surg. Glob. Open.

[16] Casella D., Bernini M., Bencini L., Roselli J., Lacaria M.T., Martellucci J., Banfi R., Calabrese C., Oryalesi L. (2014). TiLoop Bra mesh used for immediate breast reconstruction: Comparison of retropectoral and subcutaneous implant placement in a prospective single-institution series. Eur. J. Plast. Surg..

[17] Casella D., Calabrese C., Bianchi S., Meattini I., Bernini M. (2015). Subcutaneous tissue expander placement with synthetic titanium-coated mesh in breast reconstruction: Long-term results. Plast. Reconstr. Surg. Glob. Open.

[18] Marcasciano M., Kaciulyte J., Gentilucci M., Barellini L., Ribuffo D., Casella D. (2018). Skin-reduction breast reconstructions with prepectoral implant covered by a combined dermal flap and titanium-coated polypropylene mesh. J. Plast. Reconstr. Aesthet. Surg..

[19] Pagliara D., Grieco F., Schiavone L., Salgarello M., Rancati A. (2026). Breast Envelope Complications After Revision Breast Implant Surgery: A Systematic Review. Aesthetic Plast. Surg..

[20] Srinivasa D.R., Holland M., Sbitany H. (2019). Optimizing perioperative strategies to maximize success with prepectoral breast reconstruction. Gland. Surg..

[21] Sigalove S., Maxwell G.P., Sigalove N.M., Storm-Dickerson T.L., Pope N., Rice J., Gabriel A. (2017). Prepectoral implant-based breast reconstruction: Rationale, indications, and preliminary results. Plast. Reconstr. Surg..

[22] Alhumaid A.A.S., Lopez-Aguiar A., Crystal J., Oeltjen J.C., Singh D., Rojas K., Kesmodel S.B. (2025). Comparison of Surgical Complications with Direct-to-Implant vs. Tissue Expander Reconstruction After Wise Pattern Skin-Sparing Mastectomy. Eur. J. Breast Health.

[23] Komorowska-Timek E., Merrifield B., Turfe Z., Davis A.T. (2019). Subcutaneous prosthetic breast reconstructions following skin reduction mastectomy. Plast. Reconstr. Surg. Glob. Open.

[24] Spear S.L., Boehmler J.H., Bogue D.P., Mafi A.A. (2008). Options in reconstructing the irradiated breast. Plast. Reconstr. Surg..

[25] Barjot C., Gaillard T., Seban R.D., Darrigues L., Loirat D., Cabel L., Feron J.G., Fourchotte V., Couturad B., Bonneau C. (2025). Impact of neoadjuvant immunotherapy on postoperative complications in oncoplastic breast cancer surgery. Eur. J. Surg. Oncol..

[26] Rancati A.O., Angrigiani C.H., Hammond D.C., Nava M.D., Gonzalez E.G., Dorr J.C., Gercovich G., Rocco N., Rostagno R.L. (2017). Direct to implant reconstruction in nipple sparing mastectomy: Patient selection by preoperative digital mammogram. Plast. Reconstr. Surg. Glob. Open.

[27] Ran R., Wang H., He X., Li J., Yu M., Mou E., Liu C. (2025). Risk factors for complications after reduction mammaplasty: A systematic review and meta-analysis. Eur. J. Med. Res..

[28] Glasberg S.B. (2017). The economics of prepectoral breast reconstruction. Plast. Reconstr. Surg..

[29] Paganini A., Löfstrand J., Mirzaei N., Hansson E. (2026). Postmastectomy Breast Reconstruction Following Massive Weight Loss: An Updated Systematic Review and Identification of Research Gaps. Microsurgery.

[30] Casella D., Di Taranto G., Lo Torto F., Marco M., Kaciulyte J., Greco M., Onesti M.G., Ribuffo D. (2020). Body mass index can predict outcomes in direct-to-implant prepectoral breast reconstruction. Plast. Reconstr. Surg..

[31] Lee C.C., Newland M., Yau A., Chroneos R., Johnson T.S. (2025). Impact of GLP-1 Agonist on Surgical Wound Complications Following Plastic and Reconstructive Surgery: A Propensity Matched Cohort Large Database Analysis. Plast. Reconstr. Surg..

[32] Casella D., Di Taranto G., Onesti M.G., Greco M., Ribuffo D. (2019). A retrospective comparative analysis of risk factors and outcomes in direct-to-implant and two-stages prepectoral breast reconstruction: BMI and radiotherapy as new selection criteria of patients. Eur. J. Surg. Oncol..

[B33-jpm-16-00305] 33.Kong, B.H.; Abdallah, C.; Baker, J.; Muralidharan, V.J.; Arnautovic, A.; Losken, A. The Impact of Age on Outcomes Following Reduction Mammaplasty. *Ann. Plast. Surg.* **2026**, *96*, 223–227.

[34] Yanay N., Babb G., Williams-Medina E., Allbright M.L., Ogbonnah C.O., Schwarz G.S. (2026). Breast plastic surgery in perimenopausal and postmenopausal women: Menopause-informed counseling on screening, safety, and long-term breast health. Maturitas.

[35] Sullivan T., Smith J., Kermode J., McIver E., Courtemanche D.J. (1990). Rating the burn scar. J. Burn Care Rehabil..

[36] Argenta L.C., Morykwas M.J. (1997). Vacuum-assisted closure: A new method for wound control and treatment. Clinical experience. Ann. Plast. Surg..

[37] Myers M.B., Brock D., Cohn I. (1971). Prevention of skin slough after radical mastectomy by the use of a vital dye to delineate devascularized skin. Ann. Surg..

[38] Borgquist O., Ingemansson R., Malmsjö M. (2011). The influence of low and high pressure levels during negative-pressure wound therapy on wound contraction and fluid evacuation. Plast. Reconstr. Surg..

[39] Horch R.E. (2015). Incisional negative pressure wound therapy for high-risk wounds. J. Wound Care.

[40] Wilkes R.P., Kilpad D.V., Zhao Y., Kazala R., McNulty A. (2012). Closed incision management with negative pressure wound therapy (CIM): Biomechanics. Surg. Innov..

[41] Nagata T., Miura K., Homma Y., Fukamizu H. (2018). Comparison between Negative-Pressure Fixation and Film Dressing in Wound Management after Tissue Expansion: A Randomized Controlled Trial. Plast. Reconstr. Surg..

[42] Akhter H.M., Macdonald C., McCarthy P., Huang Y., Meyer B., Shostrum V.K., Cromer K.J., Johnson P.J., Wong S.L., Hon H.H. (2023). Outcomes of Negative Pressure Wound Therapy on Immediate Breast Reconstruction after Mastectomy. Plast. Reconstr. Surg. Glob. Open.

[43] Ryu J.Y., Lee J.H., Kim J.S., Lee J.S., Choi K.Y., Chung H.Y., Cho B.C., Yang J.D. (2022). Usefulness of Incisional Negative Pressure Wound Therapy for Decreasing Wound Complication Rates and Seroma Formation Following Prepectoral Breast Reconstruction. Aesthetic Plast. Surg..

[44] Irwin G.W., Boundouki G., Fakim B., Johnson R., Highton L., Myers D., Searle R., Murphy J. (2020). Negative Pressure Wound Therapy Reduces Wound Breakdown and Implant Loss in Prepectoral Breast Reconstruction. Plast. Reconstr. Surg. Glob. Open.

[45] Neron M., Delmond L., Gourgou S., Delaine S., Chalbos P., Moussion A., Taoum C. (2026). Prevention of postoperative complications with negative pressure wound therapy after complex breast cancer surgery: A study protocol of a randomised controlled trial (TPN-SEIN). BMJ Open.

[46] Webster J., Scuffham P., Stankiewicz M., Chaboyer W.P. (2014). Negative pressure wound therapy for skin grafts and surgical wounds healing by primary intention. Cochrane Database Syst. Rev..

[47] Al-Ishaq Z., Rahman E., Salem F., Taj S., Mula-Hussain L., Mylvaganam S., Vydia R., Matey P., Sircar T. (2023). Is Using Closed Incision Negative Pressure Therapy in Reconstructive and Oncoplastic Breast Surgery Helpful in Reducing Skin Necrosis?. Cureus.

[48] Gabriel A., Sigalove S.R., Maxwell G.P. (2016). Initial Experience Using Closed Incision Negative Pressure Therapy after Immediate Postmastectomy Breast Reconstruction. Plast. Reconstr. Surg. Glob. Open.

